# *Theobroma mariae*: Bioactive Compound-Rich Flowers

**DOI:** 10.3390/plants14030377

**Published:** 2025-01-26

**Authors:** Laila Y. S. Silva, Débora N. Cavalcante, Edinilze S. C. Oliveira, Andreia M. Salvador, Zilanir C. Pereira, Julia C. C. Consentini, Gabriela Furlaneto, Pedro H. Campelo, Edgar A. Sanches, Luciana Azevedo, Jaqueline de A. Bezerra

**Affiliations:** 1 Amazon Science and Technology Studies Center, Federal Institute of Education, Science and Technology of Amazonas, Manaus 69020-120, Brazil; silvalailayasmim@gmail.com (L.Y.S.S.); dnogueira14@gmail.com (D.N.C.); edinilzeoliveira@ufam.edu.br (E.S.C.O.); andreiamontoia31@gmail.com (A.M.S.); zilanircarvalho@gmail.com (Z.C.P.); 2 *In Vitro* and *In Vivo* Nutritional and Toxicological Analysis Laboratory, Federal University of Alfenas, Alfenas 37130-000, Brazil; julia.consentini@sou.unifal-mg.edu.br (J.C.C.C.); gabriela.furlaneto@sou.unifal-mg.edu.br (G.F.); luciana.azevedo@unifal-mg.edu.br (L.A.); 3 Department of Food Technology, Federal University of Viçosa, Viçosa 36570-000, Brazil; pedrocampelo@ufv.br; 4 Laboratory of Nanostructured Polymers, Materials Physics Department, Federal University of Amazonas, Manaus 69067-005, Brazil; sanchesufam@ufam.edu.br

**Keywords:** Malvaceae, phenolics compounds, food plant, NMR, LC-HRMS, citotoxicity, antiproliferative

## Abstract

Edible flowers have gained attention as unconventional food sources due to their nutritional and functional properties. This study provides novel information on the chemical composition, cytotoxicity and antiproliferative effects of *Theobroma mariae* flowers. The objective of this paper was to identify bioactive compounds in its flowers using one- and two-dimensional nuclear magnetic resonance (NMR) spectroscopy and liquid chromatography coupled with high-resolution mass spectrometry (LC-HRMS). The phenolic fraction of the flowers revealed bioactive compounds such as hyperoside, guaijaverin, astragalin, juglalin, and kaempferol. The results confirmed the potential of *T. mariae* flowers as a source of phenolic compounds, emphasizing their feasibility for possible applications in the development of functional foods. Moreover, the antiproliferative assay demonstrated that the phenolic fraction inhibits cell proliferation (GI_50_) while presenting low cytotoxicity in both cancer and normal cells.

## 1. Introduction

**Figure 1:**
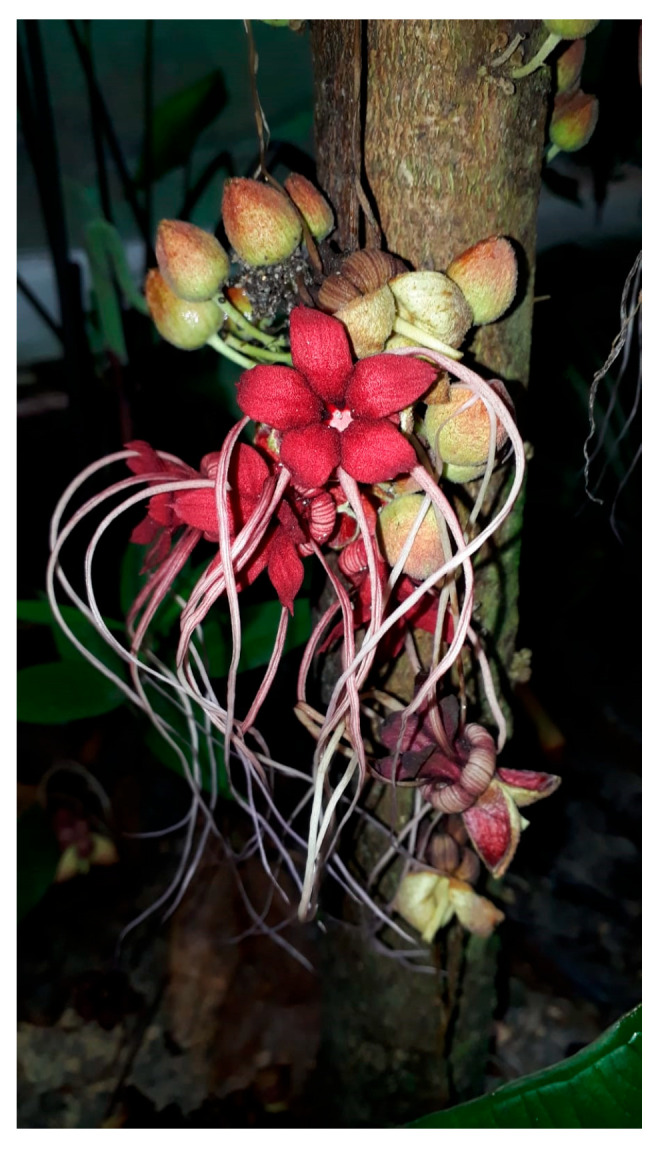
*T. mariae* flowers.

The use of natural ingredients derived from unconventional food sources, such as flowers, unripe fruits, fruit peels, plant sprouts, seeds, and rhizomes, has gained attention due to their potential for raw consumption or use in culinary preparations [1]. Currently, the genus *Theobroma* (Malvaceae) stands out for its economic value in the food, nutritional, medicinal, and artisanal sectors [2]. The *Theobroma* genus comprises 22 species distributed across the Amazon Basin and southern Mexico, among which *Theobroma cacao* L. (cocoa) and *Theobroma grandiflorum* (cupuaçu) (Willd. ex Spreng.) K. Schum.) stands out for its significant economic importance [2,3]. Chemical and pharmacological studies of extracts from these species have revealed the presence of phenolic compounds, and their consumption may provide significant health benefits, such as reducing lipid peroxidation and increasing antioxidant capacity [4]. Other species within the genus are limited to local use and remain underexplored but also present significant commercial potential [5]. Among these, we can highlight the *Theobroma mariae* (Mart.) K. Schum., commonly known as cacaurana, cacauí, cacau-carambola, cacau-jacaré, cacau-quadrado [3], cacao-cabeça-de-jacaré, and cacau-carambola [5]. The species *T. mariae* is synonymous with *Herrania mariae* (Mart.) Decne. ex Goudot [6]. It is native to the Amazon region, found in the states of Amazonas, Acre, Pará, Rondônia, and Roraima (Brazil) [7]. *T. mariae* is a small-sized plant with potential for ornamental use due to its striking bright red flowers (Figure 1) [8]. In addition, the fruit pulp has a slightly acidic flavor and is consumed fresh, as juice, or used in jam production [8]. However, scientific information about this species remains limited, with the only available data being a description of the alkaloid tetramethyl urate identified in its mature seeds [9].

In this context, we highlight the importance of conducting new chemical studies due to its potential for food applications. Accordingly, the aim of the present study was to identify the phenolic compounds in the hydroethanolic extract of *T. mariae* flowers and to assess their cytotoxicity.

## 2. Materials and Methods

### 2.1. Collection and Processing of Flower Samples

The flowers of *T. mariae* were collected at the São Sebastião site, located in the municipality of Tefé (Amazonas, Brazil) in May 2022 (coordinates 3°22′04.2″ S and 64°37′58.4″ W, SISGEN: A0B6BFD). The plant material (10.574 g) was lyophilized, resulting in 1.271 g of dry material. The flower extract was prepared following an adaptation of the method proposed by Arruda et al. [10]. The dry material (1.271 g) was subjected to extraction with an ethanol/water solution (12 mL) in an 8:2 (*v*/*v*) ratio using an ultrasonic bath (model SSBuc-6L, Mylabor, São Paulo, Brazil) at room temperature (30 min). The supernatant was then removed, and the residue underwent two additional re-extraction steps under the same conditions. The obtained extract was dried after solvent evaporation in a fume hood at room temperature. The extract yield was 30.33% (m = 385.5 mg). To obtain the fraction rich in phenolic compounds, solid-phase extraction (SPE) was performed using a SPE cartridge (Strata X 33 mm/Phenomenex, CA, USA). Before use, the SPE cartridge was conditioned by eluting 1 mL of methanol followed by 1 mL of ultrapure water. The extract, solubilized in 5 mL of ultrapure water, was loaded onto the cartridge (C = 380 mg/mL). Elution was performed successively with 1 mL of water, 5% methanol, and then with 100% methanol. The last fraction was collected in a vial and evaporated at room temperature. The last fraction was labeled phenolic fraction and subjected to further analyses: HPLC-QTOF/MS, NMR, and cytotoxicity assay.

### 2.2. Analysis by NMR

NMR analyses (^1^H, ^1^H^−13^C HSQC, and ^1^H^−13^C HMBC) of the phenolic fraction were performed on an 11.7 T spectrometer (Bruker^®^ Avance III HD 500.13 MHz for ^1^H and 125.8 MHz for ^13^C, BBFO Plus SmartProbe™, New York, NY, USA) at 298.0 K. The phenolic fraction (6.0 mg) was dissolved in 530.0 μL of CD_3_OD containing trimethylsilylpropanoic acid (TMSP) (≥99.0% purity) as the internal reference (0.0 ppm). All the processes were performed manually using the software TopSpin™ 4.1.3 (Bruker^®^).

### 2.3. Analysis by HRMS

LC-MS analysis was performed on a high-performance liquid chromatograph (HPLC) (Shimadzu^®^, Tokyo, Japan) coupled with a quadrupole time-of-flight high-resolution mass spectrometer (Q-TOF-MS) (micrOTOF- Q II, Bruker Daltonics, Fremont, CA, USA). The separation of compounds was performed using a 50 × 2.0 mm column (Shim-pack VP-ODS, 2.2 μm particle size) maintained at 35 °C. The gradient elution with a binary mobile phase consisted of water/formic acid (0.1%) (A) and acetonitrile (B). The gradient elution modes were as follows: 0–22 min (5–20% B), 22–24 min (20–100% B), 26–28 min (100–5% B), and 28–30 min (20% B). The flow was 0.4 mL min^−1^, and the sample injection volume was 5.0 μL. The capillary voltage was 3.5 kV. Nitrogen was used as a nebulizer (2.0 bar) and dry gas (6.0 L min^−1^). The mass range was considered from *m*/*z* 100 to 1000 Da. Sodium formate was used for instrument calibration. Bruker^®^ Compass Data Analysis 4.1 software was used for acquisition and processing.

### 2.4. Cytotoxicity and Antiproliferative Assay

Hep G2 (human hepatocellular carcinoma cells) and Huvec (normal human umbilical vein endothelial cells) were used in the *in vitro* experiments, obtained from the Cell Bank of Rio de Janeiro (BCRJ, RJ, Brazil). The cytotoxic effect of *T. mariae* flowers was evaluated using an MTT assay; this experiment followed the conditions and procedures previously adopted by Lima et al. [11]. MTT (3-[4,5-dimethylthiazol-2-yl]-2,5-diphenyltetrazolium bromide) is a yellow solution that is converted into blue formazan crystals by mitochondrial activity [12]. The cells were cultured in Ham’s F12 medium, supplemented with 10% (*v*/*v*) fetal bovine serum and 100 μg/mL of penicillin, and then plated in 96-well plates with 100 μL/well of culture medium, at densities of 1 × 10^4^ cells/well (Hep G2) and 6 × 10^3^ cells/well (HUVEC). After attachment, the cells were treated for 48 h with serial concentrations ranging from 25 to 500 μg/mL of the phenolic fraction of the flowers of *T. mariae*. Then, the MTT reagent (0.5 mg/mL) was added to each well, and the plates were incubated for an additional 4 h. The formazan crystals formed were dissolved in dimethyl sulfoxide (DMSO), and the absorbance was measured at 570 nm. The IC_50_ (inhibitory concentration—of the agent that inhibits 50% of cell growth), GI_50_ (growth inhibition concentration—concentration of the agent that inhibits growth by 50%), and LC_50_ (lethal concentration—concentration of the agent that results in a 50% loss of cells) were determined according to the method described by do Carmo et al. [13,14]. In addition, the selectivity index (SI) was calculated as the ratio of IC_50_ (HUVEC cell line)/IC_50_ (cancer cell line). The SI indicates the selectivity of the samples for the tested cell lines, and according to do Carmo et al. [14], values of the SI greater than 3 are considered indicative of high selectivity [14].

### 2.5. Qualitative Chemical Description

**Figure 2:**
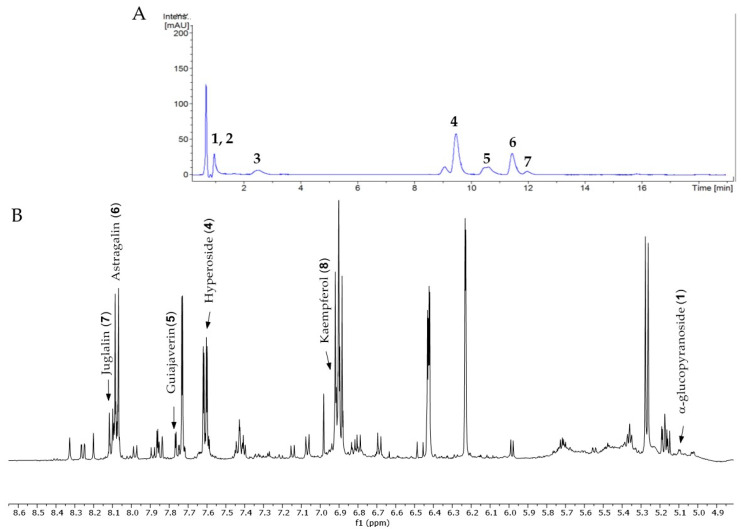
(**A**) Chromatogram of phenolic fraction of *T. mariae* flowers (190–400 nm). (**B**) Amplification of the ^1^H NMR spectra (5.0–8.6 ppm) for the signals of the compounds identified in the phenolic fraction of *T. mariae* flowers (500.13 MHz, CD_3_OD). Peak numbers correspond to those of Table 1. (**2**): malic acid e (**3**) Unknown.

The phenolic fraction of *T. mariae* flowers was analyzed using the HPLC-ESI-QTOF-MS technique (negative mode). The resulting chromatogram is shown in Figure 2A, and the identified constituents are presented according to their retention times (RT 0–20.6 min, Table 1). Seven peaks were observed in the phenolic fraction, and the constituents were identified by interpreting the experimental and theoretical *m*/*z*, fragmentation patterns, molecular formulas, and errors (in ppm) taking into account literature information [1,2,15,16,17,18,19]. The peak at 0.9 min with an *m*/*z* of 133.0134 [M−H]^−^ (error −6.37 ppm), corresponding to the molecular formula C_4_H_5_O_5_−, was attributed to malic acid (**2**), which was previously identified in the flowers of *T. speciosum* [1]. The peaks 4 (RT 9.5 min) and 5 (RT 10.6 min) exhibited base peak ions in the MS2 spectra at *m*/*z* 300.0275 [M−163−H]^−^ and *m*/*z* 300.0246 [M−133−H]^−^, respectively, suggesting the presence of the aglycone quercetin. Meanwhile, peak 6 (RT 11.6 min) showed a precursor ion at *m*/*z* 447.0927 [M−H]^−^ (C_20_H_19_O_11_), and its MS/MS spectrum displayed a fragment ion at *m*/*z* 284.0293 [M−H]^−^ (100% relative abundance), indicating the loss of a hexose unit (163 Da) through homolytic cleavage.

Additionally, peak 7 (RT 12.1 min) exhibited an ion at *m*/*z* 417.0835 [M−H]^−^ (C_20_H_17_O_10_), generating a fragment ion at *m*/*z* 284.0347 [M−H]^−^, corresponding to a loss of 133 Da, which is consistent with an arabinopyranoside unit. The constituents were identified in conjunction with the data from 1D and 2D NMR analysis (^1^H, HSQC, and HMBC), taking into account literature information. The ^1^H NMR spectral profile showed characteristic signals in three different regions: aliphatic signals (0.7 to 3.0 ppm), followed by signals in the carbinolic region (3.0 to 5.5 ppm), and aromatic/vinyl hydrogen signals (6.0 to 8.3 ppm, Figure 2B). In the carbohydrate region, the presence of α-glucopyranoside (**1**) was detected by the signal at δ_H_ 5.08 (d; *J* = 3.7 Hz, H-1). In the aromatic region, characteristic signals were observed, compatible with the typical aromatic substitution pattern of flavonoid structures, with meta spin coupling at δ_H_ 6.23 (d; *J* = 2.0 Hz, H-6) and δ_H_ 6.42 (d; *J* = 2.0 Hz, H-8) (ring A), as well as signals compatible with an ortho-, ortho–meta-, and meta-coupling pattern (ring B). The flavonol hyperoside (**4**) was confirmed by signals at δ_H_ 7.73 (d, *J* = 2.1 Hz, H-2′), δ_H_ 6.80 (d, *J* = 8.1 Hz, H-5′), and a doublet of doublets at δ_H_ 7.61 (d, *J* = 8.1; 2.1 Hz), consistent with the 3′,4′-dioxygenated B-ring system [1,15]. The flavonoid guaijaverin (**5**) was characterized by resonances at δ_H_ 7.76 (d, *J* = 2.1 Hz, H-2′) and δ_H_ 7.60 (d, *J* = 8.4; 2.1 Hz) [2,15]. Additionally, characteristic signals of astragalin (**6**) were observed at δ_H_ 8.07 (d, *J* = 8.9 Hz, H-2′, H-6′) and δ_H_ 6.89 (d, *J* = 8.9 Hz, H-3′, H-5′) [16]. Furthermore, the presence of two doublets at δ_H_ 8.10 (d, *J* = 8.9 Hz, H-2′, H-6′) and δ_H_ 6.89 (d, *J* = 8.9 Hz, H-3′, H-5′) indicates the ortho–meta coupling attributed to the compound juglalin (**7**) [17]. The same coupling pattern was observed at δ_H_ 8.08 (d, *J* = 8.8 Hz, H-2′, H-6′) and δ_H_ 6.90 (d, *J* = 8.8 Hz, H-3′, H-5′), assigned to the flavonol kaempferol (**8**) [18,19]. All chemical descriptions of the flavonoids identified in the phenolic fraction are detailed in Table 1. Among these, the constituent (**4**) has already been reported in the seeds of *T. grandiflorum* [20]. The compounds (**4**) and (**5**) have also been reported in the flowers of *T. speciosum* [1] and chocolate samples from *T. cacao* [21]. This is the first report of phenolic compounds identified in the flowers.

### 2.6. Cytotoxic and Antiproliferative Assay of T. mariae Flowers

**Figure 3:**
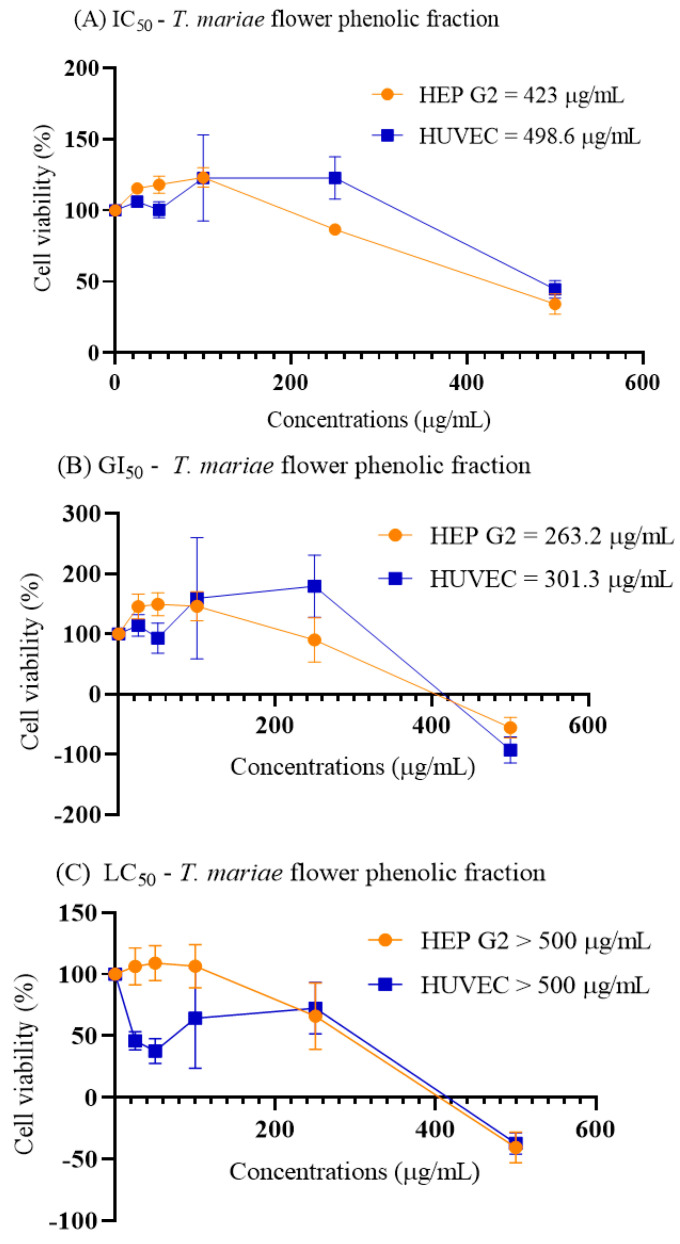
Cytotoxic activity of the phenolic fraction of *T. mariae* flower on Hep G2 and HUVEC cell lines. (**A**) IC_50_ is the concentration at which cell growth is inhibited by 50%. (**B**) GI_50_ represents the concentration that reduces cell proliferation by 50% when compared to control cells. (**C**) LC_50_ is the concentration at which there is a 50% reduction in the cell population compared to the initial cell count at the start of the treatment.

Based on the cytotoxicity classification by Anywar et al. [22], the results indicated that the phenolic fraction of *T. mariae* flowers exhibited weak cytotoxicity against Hep G2 and HUVEC cells, with IC_50_ values of 423 μg/mL and 498.6 μg/mL, respectively, and low selectivity for cancer cells (SI = 1.18) (Figure 3).

Additionally, the phenolic fraction demonstrated antiproliferative activity across all tested cell lines, with GI_50_ values of 263.2 μg/mL for Hep G2 and 301.3 μg/mL for HUVEC. Another species within the *T. mariae* family (Malvaceae), *Sida santaremnensis* H. Monteiro, has similarly shown notable antiproliferative activity in cellular models, attributed to the flavonoid kaempferol (**8**) [23]. This flavonoid has been proposed for the treatment of hepatocellular carcinoma due to its antitumor properties [24,25]. Additionally, it has been demonstrated that the glycosylated flavonoid hyperoside (**4**) inhibits the proliferation of hepatocellular carcinoma cells (Hep G2) [26] and induces apoptosis in breast [27], ovarian [28], and lung cancer cells [29]. Studies on the flavonoid astragalin (**6**) suggest that its anticancer effect is attributed to its ability to inhibit the proliferation of three different hepatocellular carcinoma cell lines (Hep G2, Huh-7, and H22) *in vitro* [30]. Furthermore, astragalin has been shown to prevent alcohol-induced acute liver injury in mice, reduce lipid peroxidation, and enhance antioxidant activity [31]. These compounds identified in our chemical analyses (Table 1), may contribute directly to the observed cellular effects. Moreover, the phenolic fraction was not lethal (LC_50_) at the tested concentrations, demonstrating safe parameters. In summary, the phenolic fraction inhibits cell proliferation (GI_50_) and exhibits weak cytotoxicity in both cancer and normal cells, without inducing cell death. These findings pave the way for future research on this phenolic fraction.

## 3. Conclusions

Seven constituents, including five flavonoids, were identified in the phenolic fraction of *T. mariae* flowers, marking the first report of these compounds in this species. Additionally, the phenolic fraction exhibited antiproliferative activity against both normal and cancer cell lines. These findings provide a valuable foundation for future chemical and pharmacological research on extracts or bioactive products derived from *T*. *mariae*, highlighting its potential for therapeutic applications.

## Figures and Tables

**Table 1:** Compounds identified in of *Theobroma mariae* phenolic fraction by HPLC-ESI-QTOF-MS/MS (negative mode) and NMR (^1^H and ^13^C, CD_3_OD).

No^o^	RT (Min)	Compounds Structure	[M−H]^−^ Calculated	[M−H]^−^ Observed (ion Formula, Error in ppm)	Fragmentation	1H in ppm (* Multiplicity; *J* in Hz, H)	^13^C in ppm	References
1	0.8	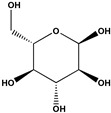 α-glucopyranoside	179.055563	179.0552 (C_6_H_11_O_6_, −2.03)	–	5.08 (d; *J* = 3.7 Hz, H-1).	–	[1]
2	0.9	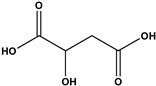 Malic acid	133.014247	133.0134 (C_4_H_5_O_5_, −6.37)	–	–	–	[1]
3	2.6	Unknown	188.034768	188.0340 (C_10_H_6_NO_3_, −4.08)	144.0468 (100.0%)	–	–	
4	9.6	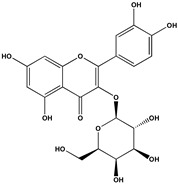 Quercetin-3-galactoside (Hyperoside)	463.088200	463.0877 (C_21_H_19_O_12_, −1.08)	300.0275 (100.0%)	6.23 (d; *J* = 2.0 Hz, H-6), 6.42 (d; *J* = 2.0 Hz, H-8), 7.73 (d; *J* = 2.2 Hz, H-2′), 6.80 (d; *J* = 8.5 Hz, H-5′), 7.61 (dd; *J* = 8.5, 2.2 Hz, H-6′), 5.15 (d; *J* = 7.7 Hz, H-1″).	157.6 (C-2), 134.4 (C-3), 161.3 (C-5) 98.6 (C-6), 164.5 (C-7), 93.4 (C-8), 104.4 (C-10), 116.1 (C-2′), 144.2 (C-3′), 148.5 (C-4′), 114.8 (C-5′), 121.8 (C-6′), 103.4 (C-1″).	[1,15]
5	10.6	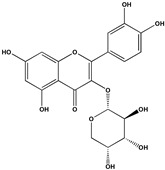 Quercetin-3-*O*-arabinopyranoside (Guaijaverin)	433.077635	433.0777 (C_20_H_17_O_11_, 0.15)	300.0246 (100.0%) 301.0338 (35.6%)	7.76 (d; *J* = 2.1 Hz, H-2′), 7.60 (dd; *J* = 8.4, 2.1 Hz, H-6′), 5.16 (d; *J* = 6.2 Hz, H-1″).	157.6 (C-2), 116.1 (C-2′), 148.5 (C-4′), 121.8 (C-6′), 103.4 (C-1″).	[2,15]
6	11.6	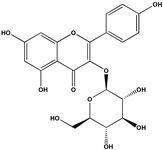 Kaempferol-3-*O*-glucoside (Astragalin)	447.092736	447.0927 (C_20_H_19_O_11_, −0.08)	284.0293 (100.0%) 285.0395 (39.0%)	6.23 (d; *J* = 2.0 Hz, H-6), 6.42 (d; *J* = 2.0 Hz, H-8), 8.07 (d; *J* = 8.9 Hz, H-2′, H-6′), 6.89 (d; *J* = 8.9 Hz, H-3′, H-5’), 5.27 (d, *J* = 7.5 Hz, H-1”).	159.8 (C-2), 134.2 (C-3), 161.3 (C-5) 98.6 (C-6), 164.5 (C-7), 93.4 (C-8), 104.4 (C-10), 121.5 (C-1’), 130.8 (C-2’, C-6’), 114.8 (C-3’, C-5’), 103.7 (C-1”).	[16]
7	12.1	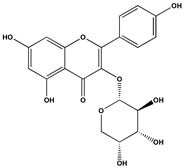 Kaempferol-3-*O*-arabinopyranoside (Juglalin)	417.082720	417.0835 (C_20_H_17_O_10_, 1.87)	284.0347 (100.0%) 285.0363 (36.6%)	8.10 (d; *J* = 8.9 Hz, H-2′, H-6’), 6.89 (d; *J* = 8.9 Hz, H-3’, H-5’).	159.8 (C-2), 121.5 (C-1’), 130.8 (C-2’, C-6’), 114.8 (C-3’, C-5’).	[17]
8	20.6	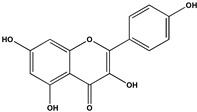 Kaempferol	285.039364	285.0391 (C_15_H_9_O_6_, -0.93)	–	8.08 (d; *J* = 8.8 Hz, H-2’, H-6’), 6.90 (d; *J* = 8.8 Hz, H-3’, H-5’).	159.8 (C-2), 121.5 (C-1’), 114.8 (C-3’, C-5’), 130.8 (C-2’, C-6’),	[18,19]

* Multiplicity: d, doublet; dd, doublet of doublets.

## Data Availability

All data are provided within the article and its Supplementary Materials.

## Supplementary Materials

The following Supporting Information can be downloaded at: https://www.mdpi.com/article/10.3390/plants14030377/s1, Figures S1 and S2: ^1^H NMR spectra of the phenolic fraction of *Theobroma mariae* (500.13 MHz, CD_3_OD); Figures S3–S5: ^1^H^−13^C HSQC of phenolic fraction of *T. mariae* (11.74 T, CD_3_OD). Figures S6–S8: ^1^H^−13^C HMBC of phenolic fraction of *T. mariae* (11.74 T, CD_3_OD). Figures S9–S17: Chromatogram and negative ion ESI-MS spectra of phenolic fraction of *T. mariae* flowers.
