# Supplementary material for: Theobroma mariae: Bioactive Compound-Rich Flowers
Source: Plants (Basel). 2025 Jan 26;14(3):377. doi: 10.3390/plants14030377 (PMC11820393; doi:10.3390/plants14030377)

SUPPLEMENTARY MATERIAL

Communication

Theobroma mariae: Bioactive Compounds-Rich Flowers

Laila Y. S. Silva [1], Débora N. Cavalcante [1], Edinilze S. C. Oliveira [1], Andreia M. Salvador [1], Zilanir C. Pereira [1], Julia C. C. Consentini [2], Gabriela Furlaneto [2], Pedro H. Campelo [3], Edgar A. Sanches [4], Luciana Azevedo [2] and Jaqueline A. Bezerra [1,*]

[1] Amazon Science and Technology Studies Center, Federal Institute of Education, Science and Technology of Amazonas, Manaus 69020-120, Brazil; silvalailayasmim@gmail.com, dnogueira14@gmail.com, edinilzeoliveira@ufam.edu.br, andreiamontoia31@gmail.com, zilanircarvalho@gmail.com,
[2] *In vitro* and *in vivo* Nutritional and Toxicological Analysis Laboratory, Federal University of Alfenas, Alfenas, Minas Gerais, Brazil; julia.consentini@sou.unifal-mg.edu.br, gabriela.furlaneto@sou.unifal-mg.edu.br, luciana.azevedo@unifal-mg.edu.br,
[3] Department of Food Technology, Federal University of Viçosa, Viçosa 36570-000, Brazil; pedrocampelo@ufv.br
[4] Laboratory of Nanostructured Polymers, Materials Physics Department, Federal University of Amazonas, Manaus 69067-005, Brazil; sanchesufam@ufam.edu.br
* Correspondence: jaqueline.araujo@ifam.edu.br

[1] Amazon Science and Technology Studies Center, Federal Institute of Education, Science and Technology of Amazonas, Manaus 69020-120, Brazil; silvalailayasmim@gmail.com, dnogueira14@gmail.com, edinilzeoliveira@ufam.edu.br, andreiamontoia31@gmail.com, zilanircarvalho@gmail.com,
[2] *In vitro* and *in vivo* Nutritional and Toxicological Analysis Laboratory, Federal University of Alfenas, Alfenas, Minas Gerais, Brazil; julia.consentini@sou.unifal-mg.edu.br, gabriela.furlaneto@sou.unifal-mg.edu.br, luciana.azevedo@unifal-mg.edu.br,
[3] Department of Food Technology, Federal University of Viçosa, Viçosa 36570-000, Brazil; pedrocampelo@ufv.br
[4] Laboratory of Nanostructured Polymers, Materials Physics Department, Federal University of Amazonas, Manaus 69067-005, Brazil; sanchesufam@ufam.edu.br
* Correspondence: jaqueline.araujo@ifam.edu.br

[*]Corresponding author, e-mail: jaqueline.araujo@ifam.edu.br

ORCID

Jaqueline de Araújo Bezerra https://orcid.org/0000-0002-9168-9864

Analysis by Nuclear Magnetic Resonance spectroscopy 1D and 2D

Figure S1

^{1}H NMR spectra of the phenolic fraction of *Theobroma mariae* (500.13 MHz, CD_3OD).

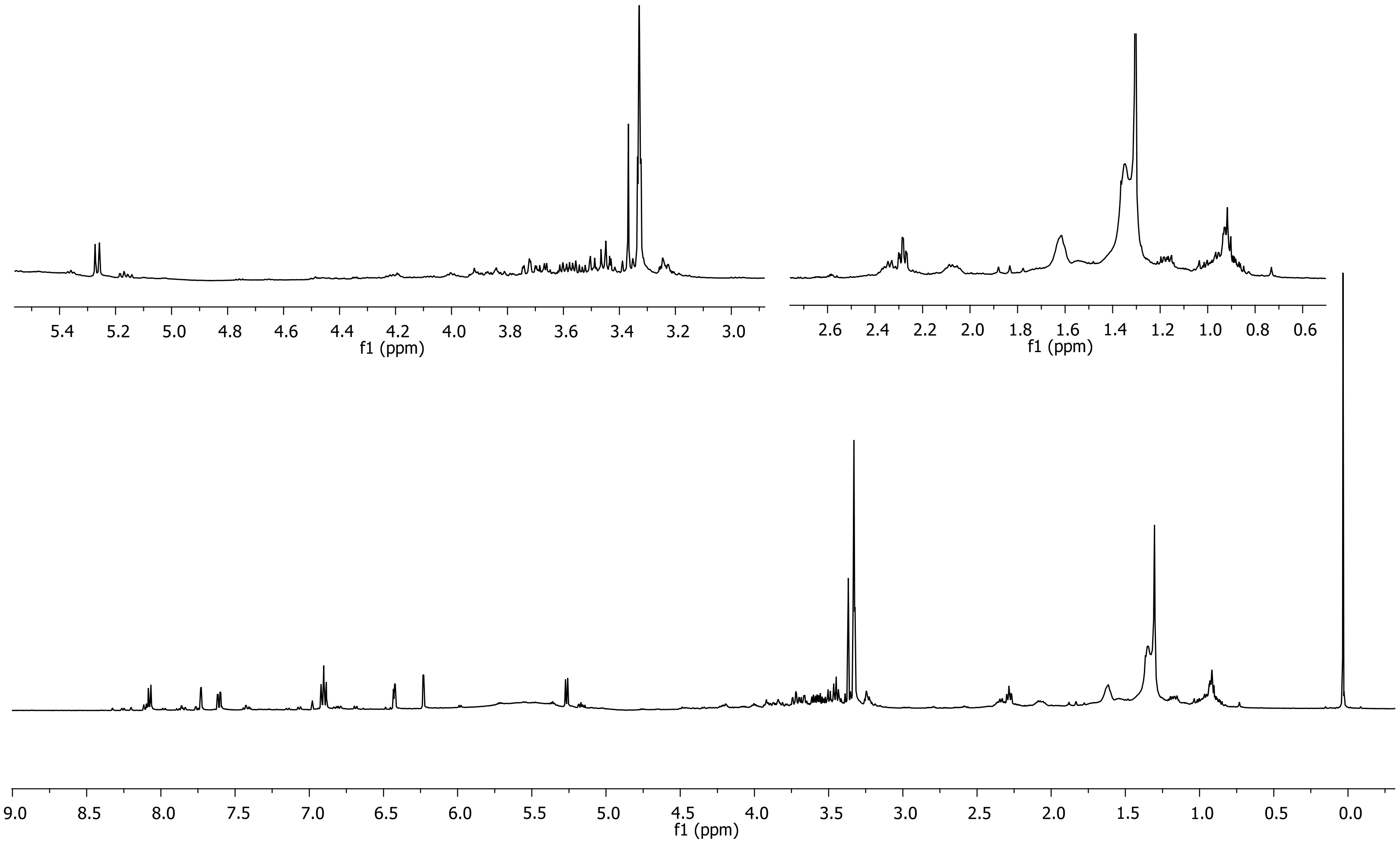

Figure S2

Amplification of the ^{1}H NMR spectra (6.0-8.3 ppm) for the signals of the compounds identified in the phenolic fraction of *T. mariae* flowers (500.13 MHz, CD_3OD)

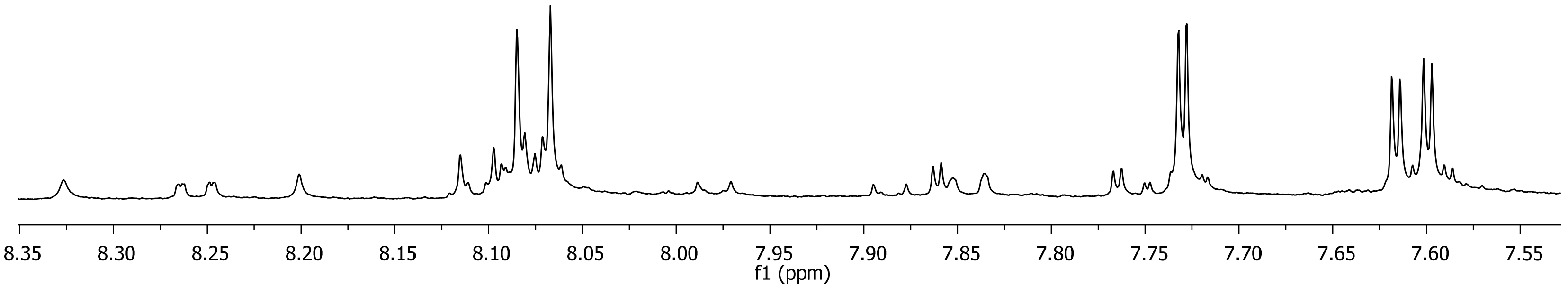

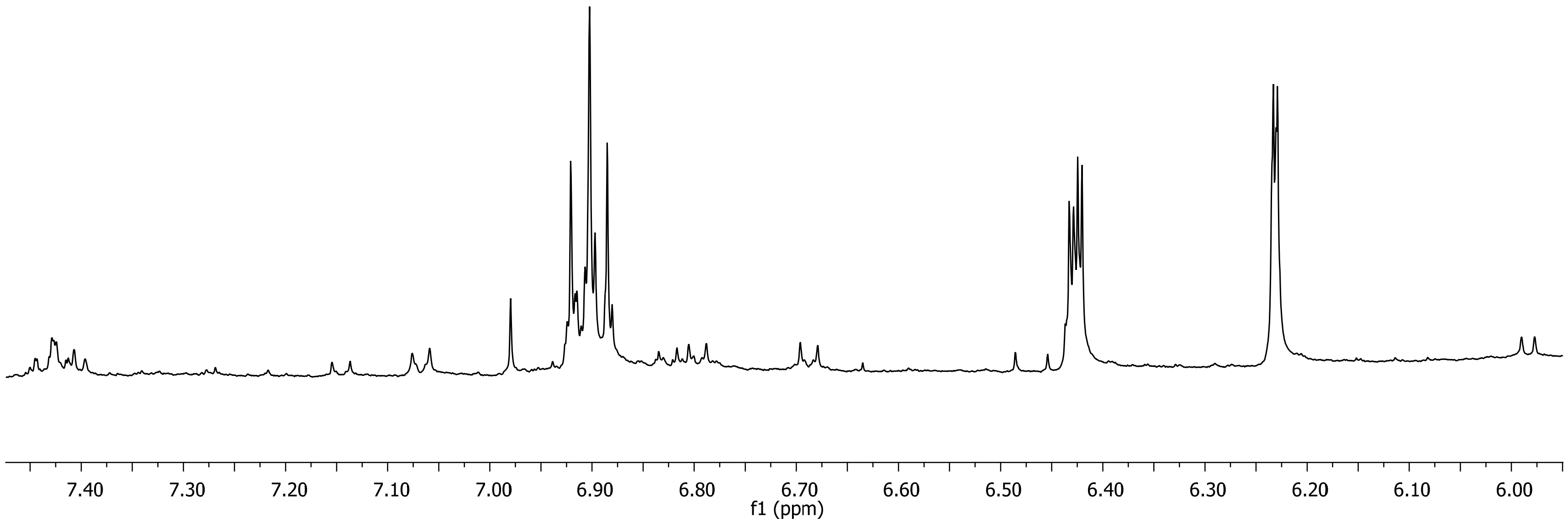

Figure S3

^{1}H – ^{13}C HSQC of the phenolic fraction of *T. mariae* (11.74 T, CD_3OD).

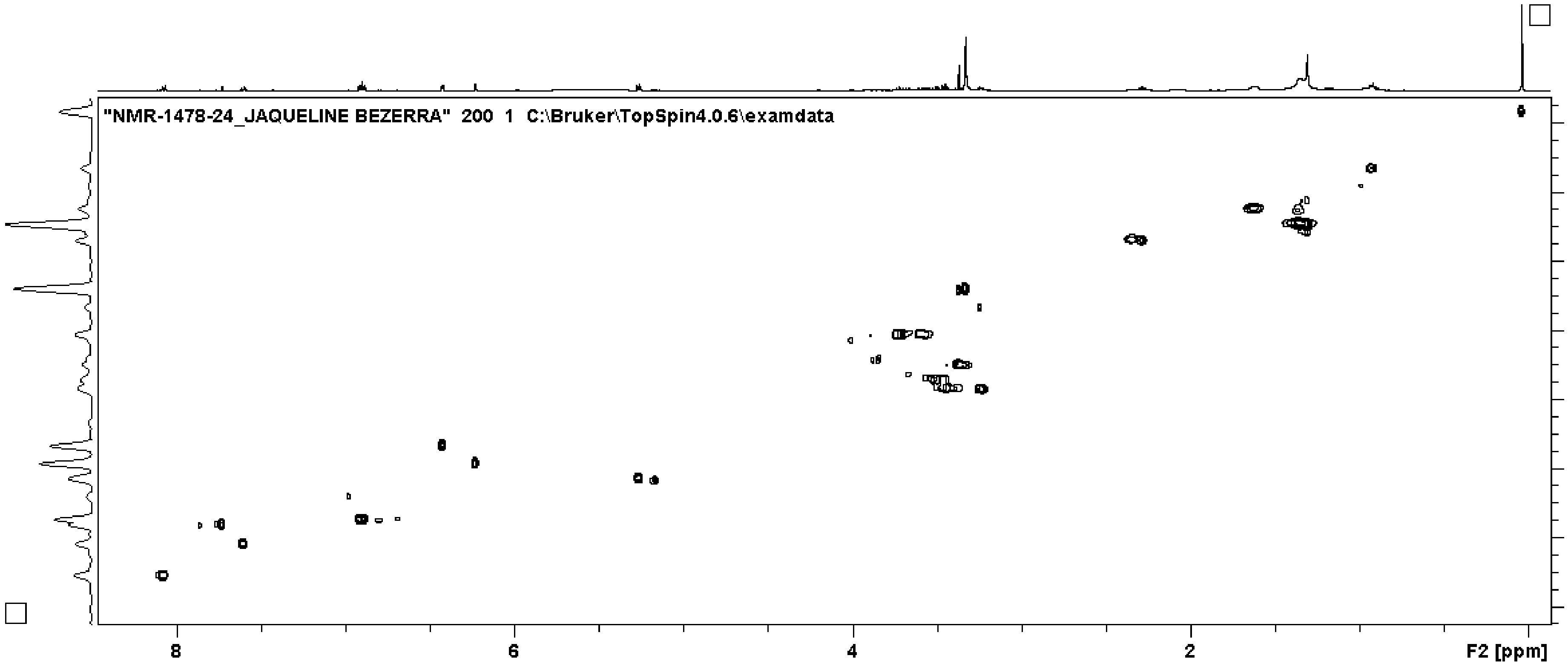

Figure S4

Amplification of the ^{1}H – ^{13}C HSQC of the phenolic fraction of *T. mariae* (11.74 T, CD_3OD).

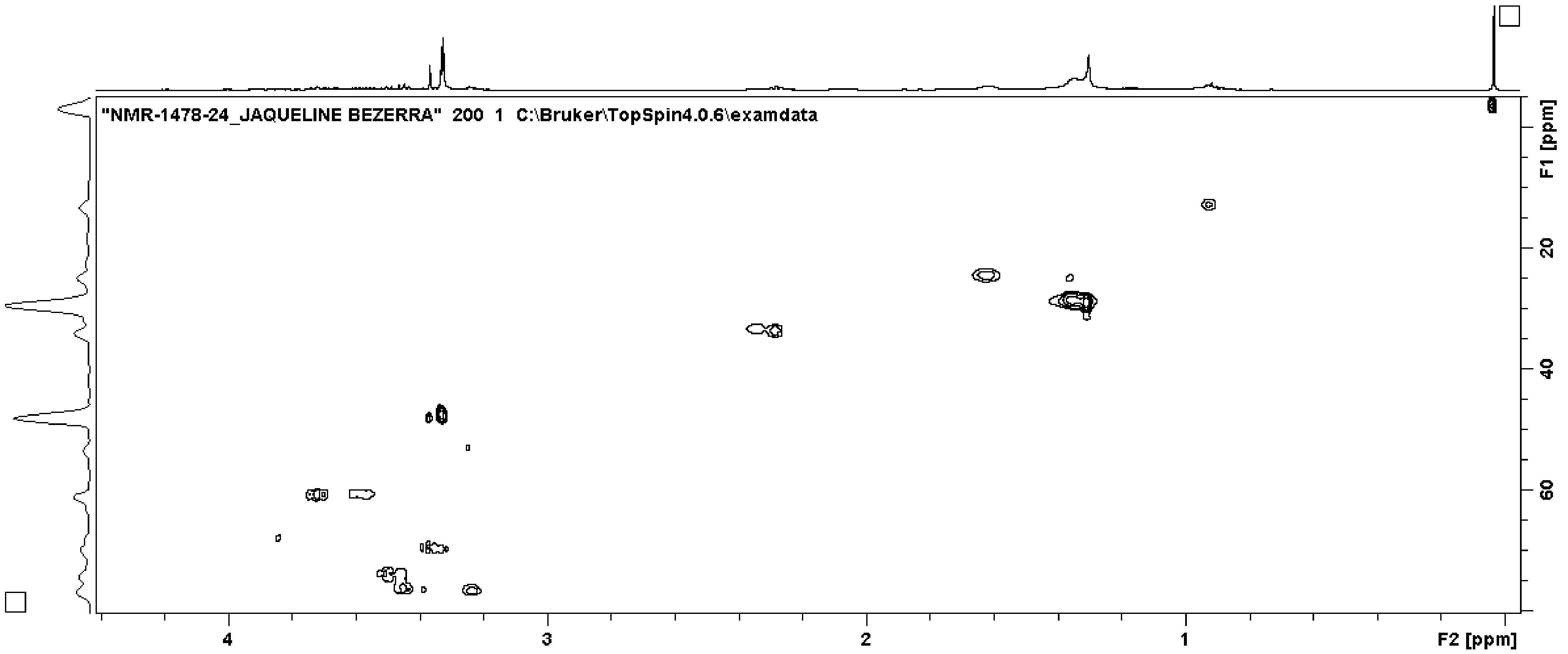

Figure S5

Amplification of the ^{1}H – ^{13}C HSQC of the phenolic fraction of *T. mariae* (11.74 T, CD_3OD).

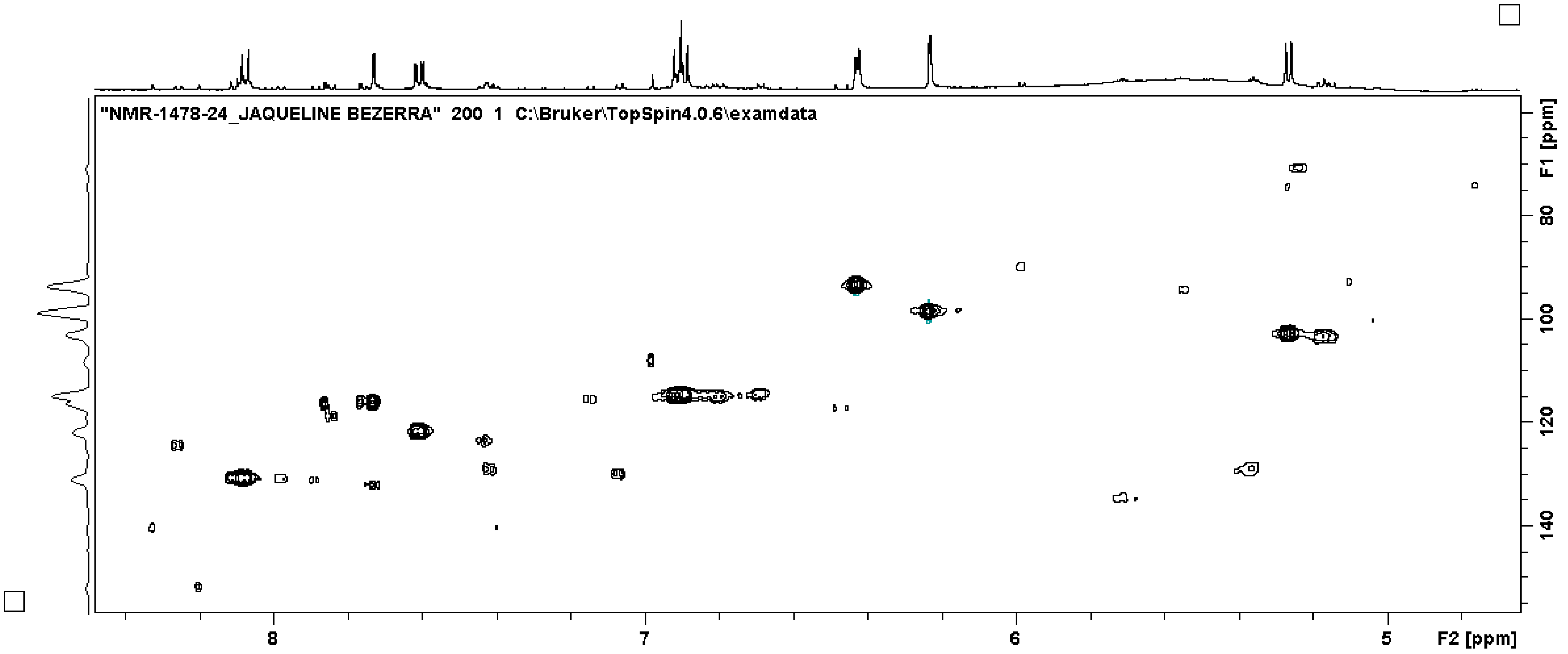

Figure S6

^{1}H – ^{13}C HMBC of the phenolic fraction of *T. mariae* (11.74 T, CD_3OD).

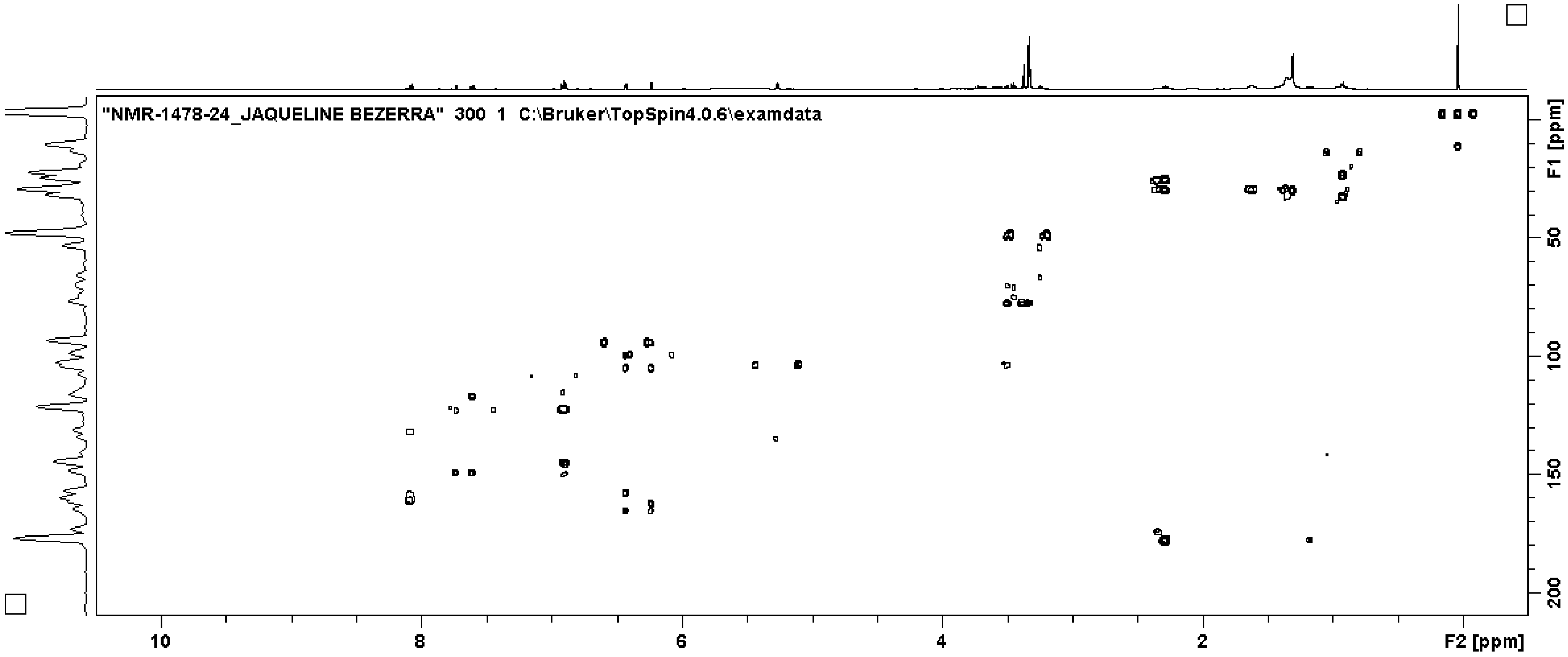

Figure S7

Amplification of the ^{1}H – ^{13}C HMBC of the phenolic fraction of *T. mariae* (11.74 T, CD_3OD).

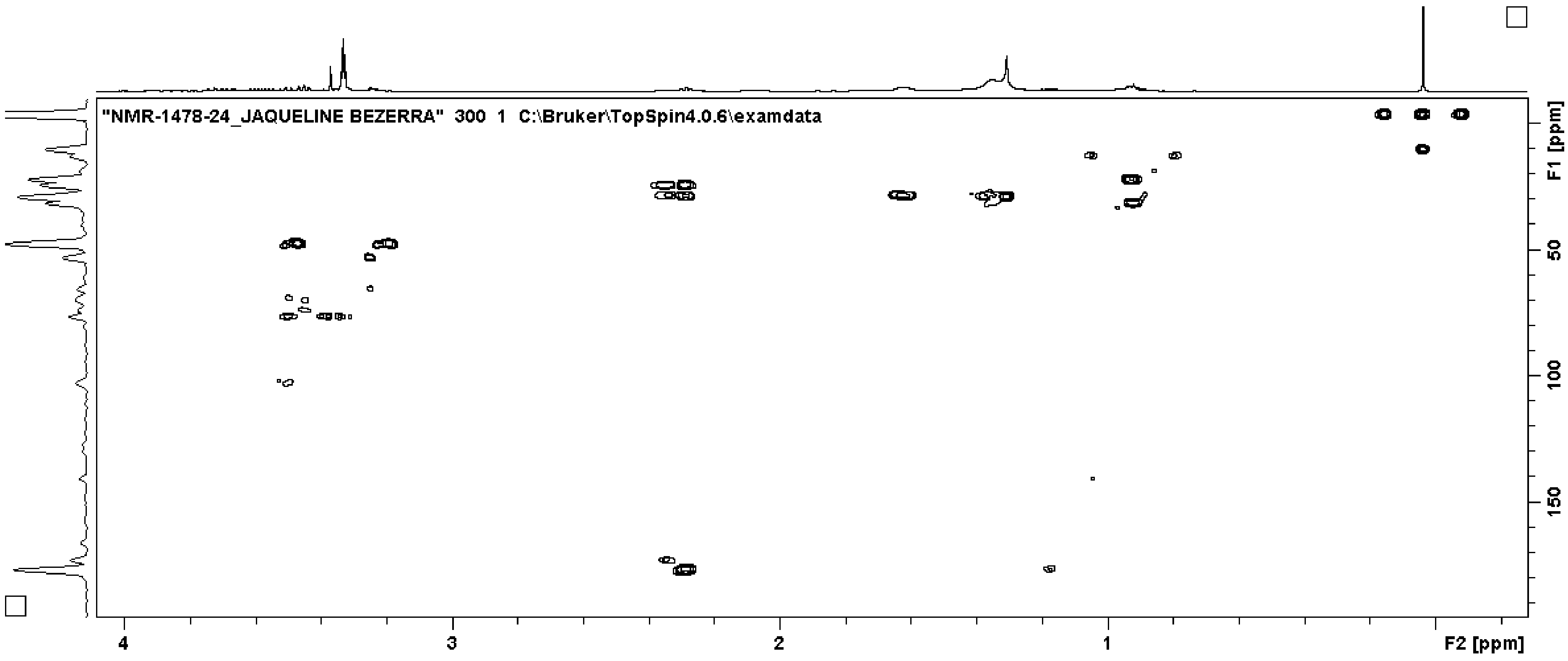

Figure S8

Amplification of the ^{1}H – ^{13}C HMBC of the phenolic fraction of *T. mariae* (11.74 T, CD_3OD).

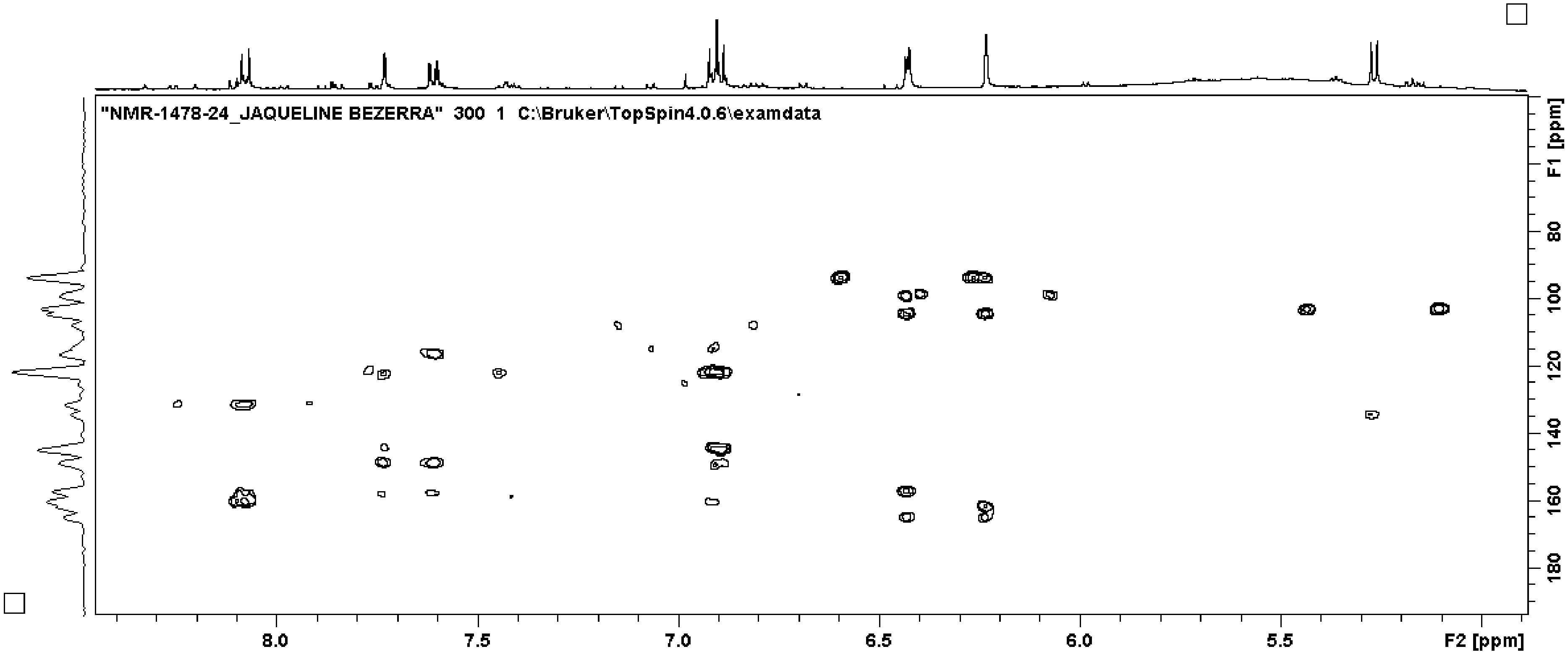

Figure S9
Chromatogram of phenolic fraction of *T. mariae* flowers by HPLC-ESI-HRMS in negative mode.

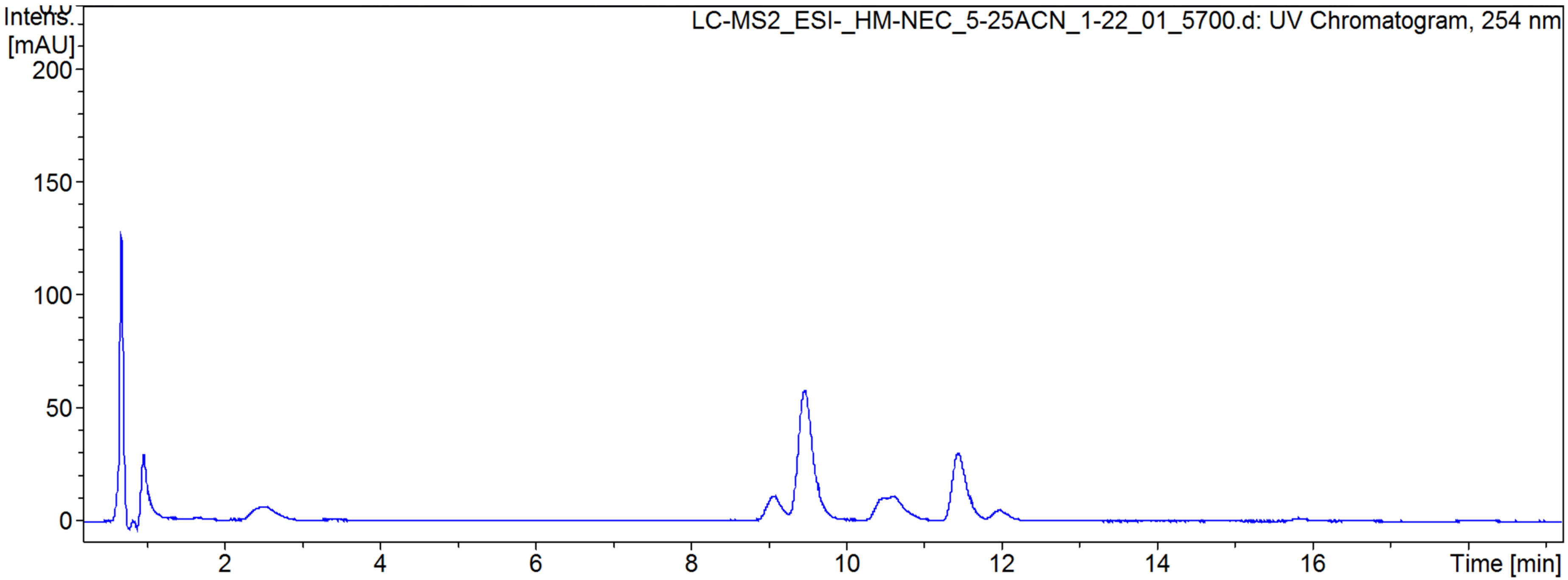

Figure S10

Peak at RT 9.1-9.2 min, negative ion ESI-MS spectra of *m/z* 927.1856.

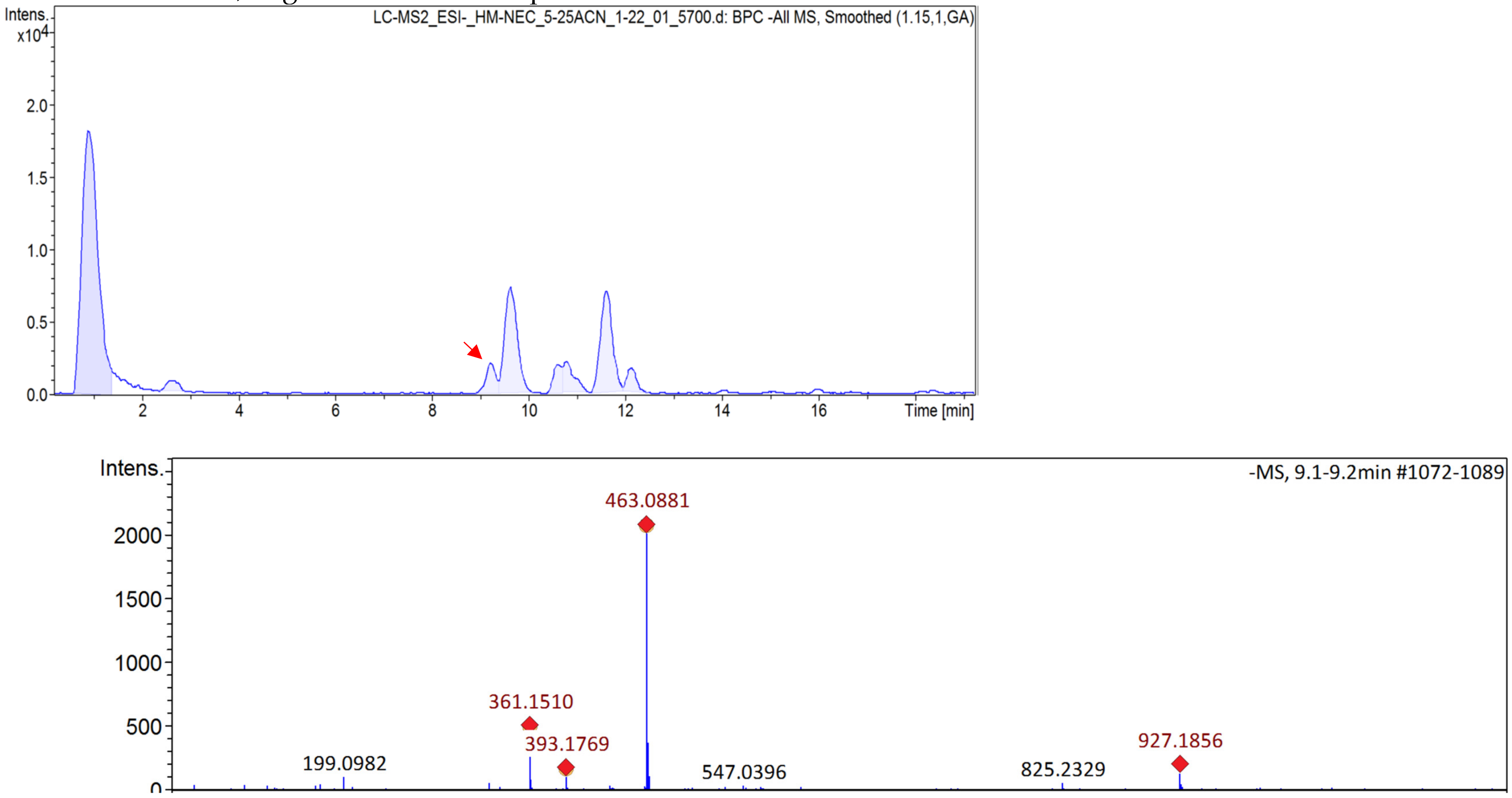

At RT 9.1–9.2 min, a high-intensity ion with m/z 463.0881 $[M-H]^-$ and a low-intensity ion with m/z 927.1856 $[2M-H]^-$ were detected (Figure S9). This finding indicates that quercetin-3-galactoside underwent dimerization during ionization. Consequently, the peak was not included in the chromatogram presented in the manuscript (Figure 2A), as the ion formed is not endogenous to the sample.

Figure S11

Peak at RT 0.7-1.1 min, negative ion ESI-MS spectra of *m/z* 179.0552 (α-glucopyranoside) and 133.0134 (malic acid).

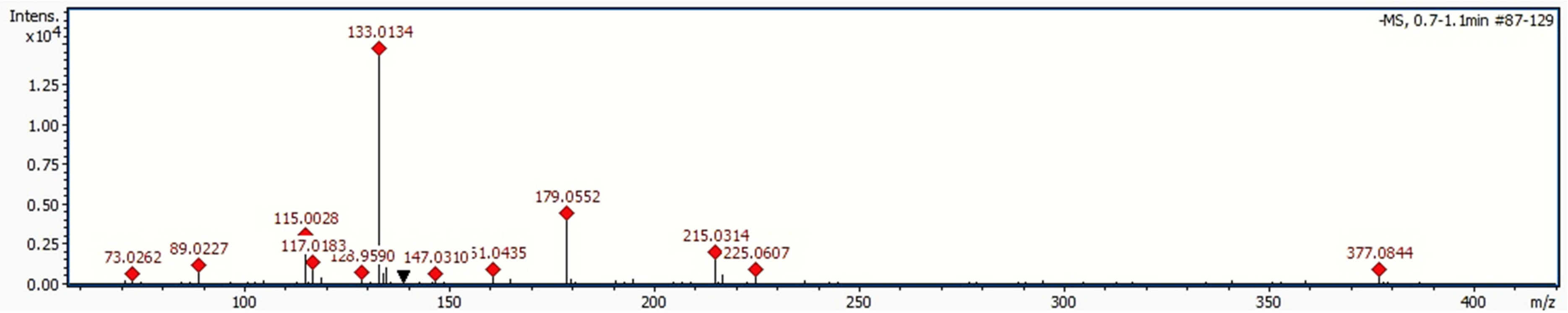

Figure S12

Peak at RT 2.6 min, negative ion ESI-MS spectra and MS^2 of *m/z* 188.0340 (Unknown)

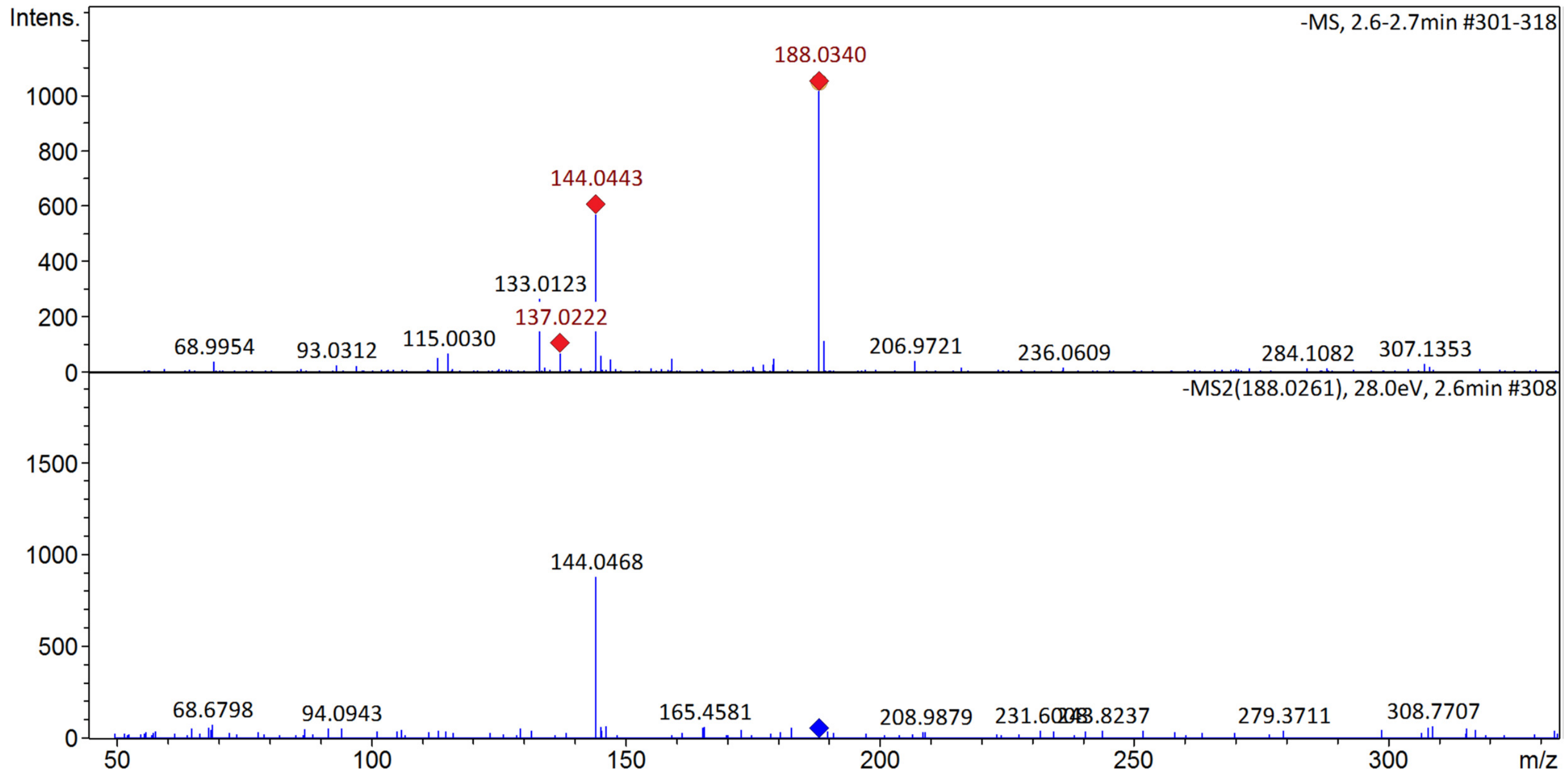

Figure S13

Peak at RT 9.6 min, negative ion ESI-MS spectra and MS^2 of *m/z* 463.0877 (Hyperoside, **4**)

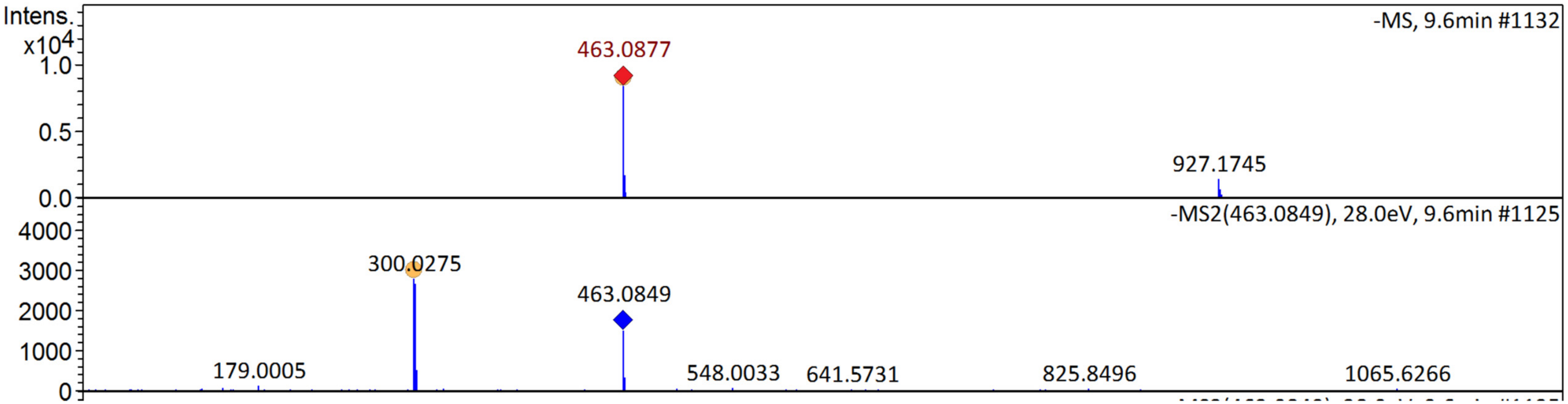

Figure S14

Peak at RT 10.6 min, negative ion ESI-MS spectra and MS^2 of *m/z* 433.0777 (Guaijaverin, **5**).

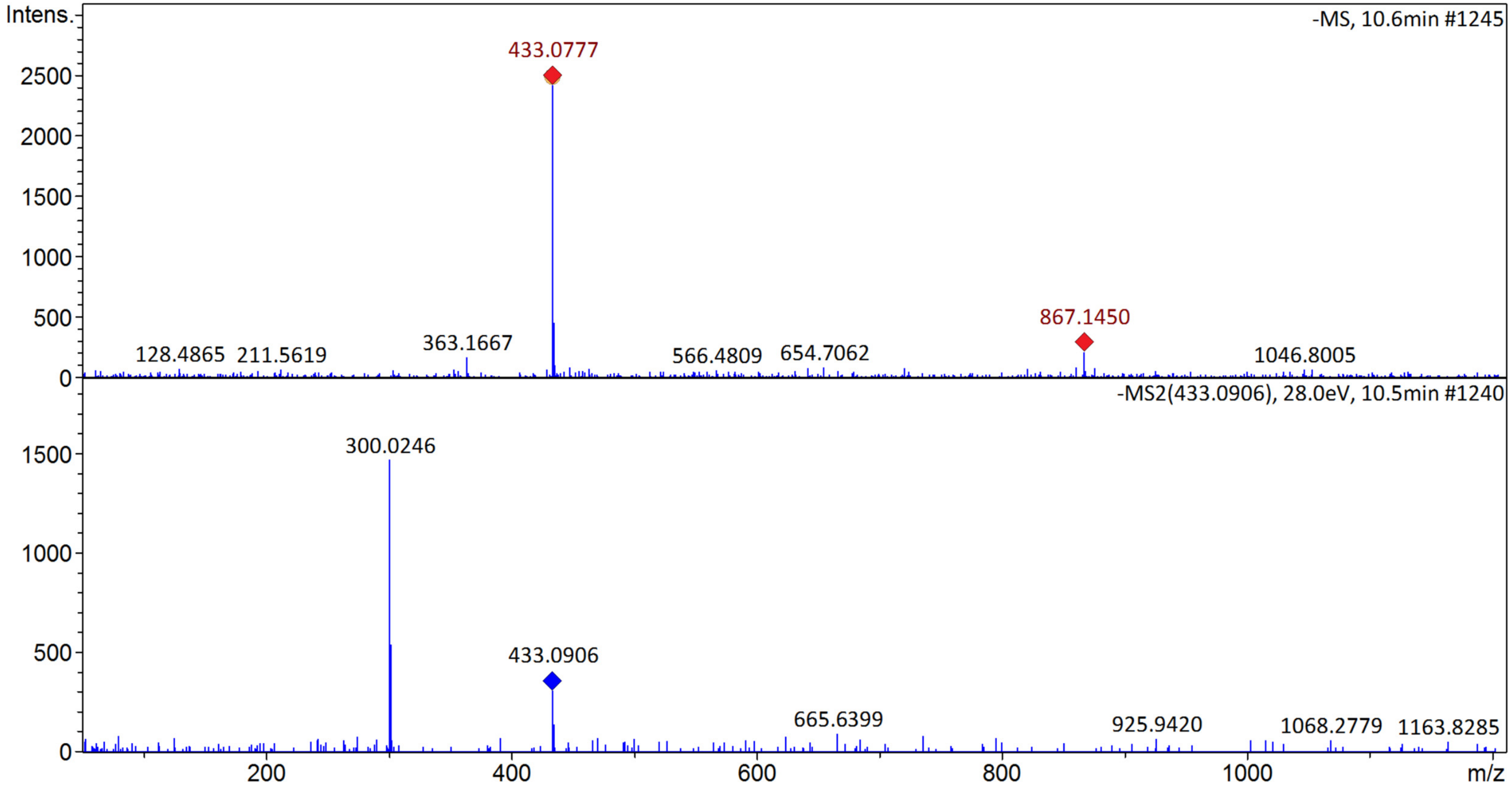

Figure S15
Peak at RT 11.6 min, negative ion ESI-MS spectra and MS^2 of *m*/*z* 447.0927 (Astragalin, **6**).

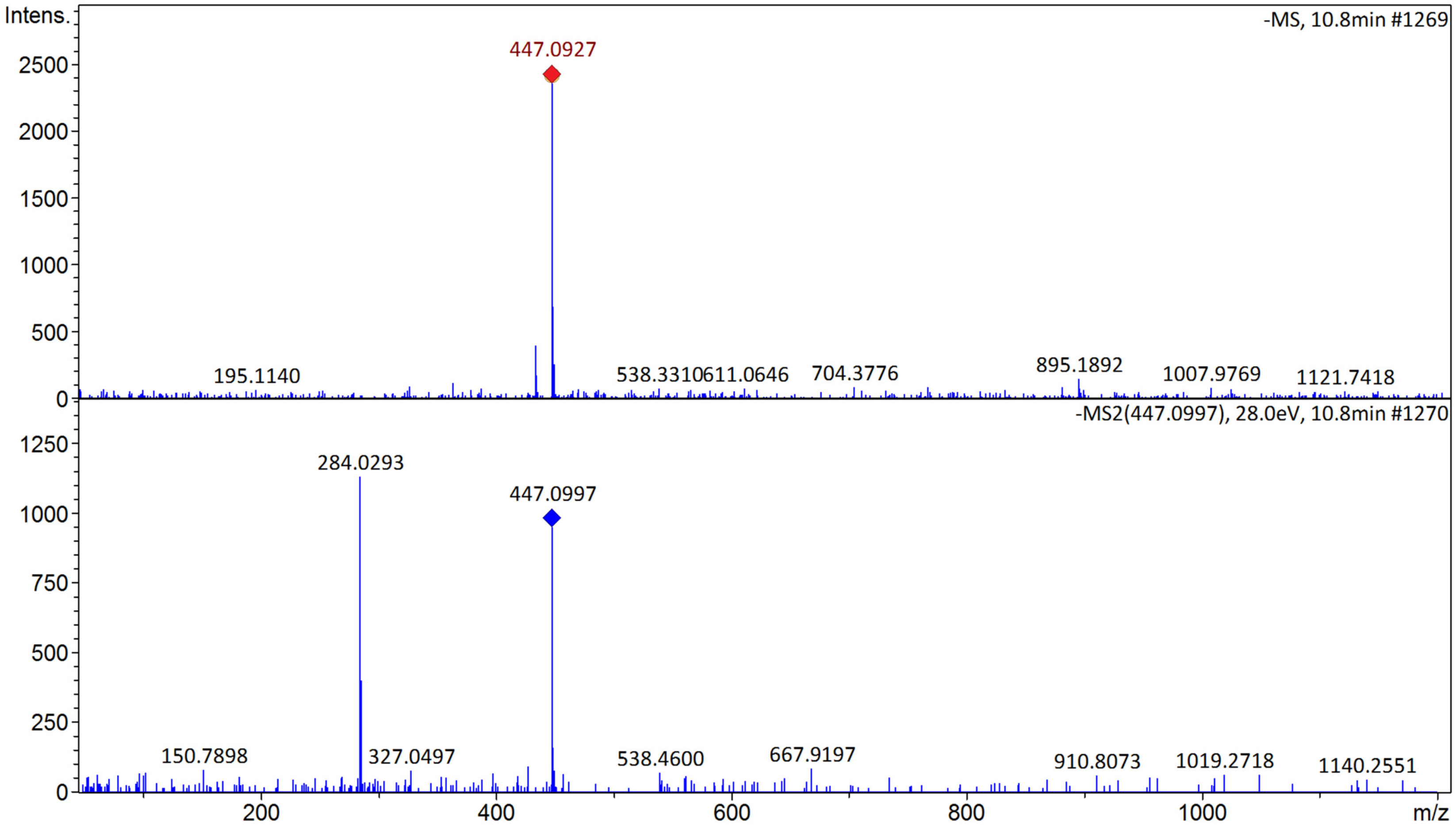

Figure S16
Peak at RT 12.1 min, negative ion ESI-MS spectra and MS[2] of *m/z* 417.0835 (Juglalin, **7**).

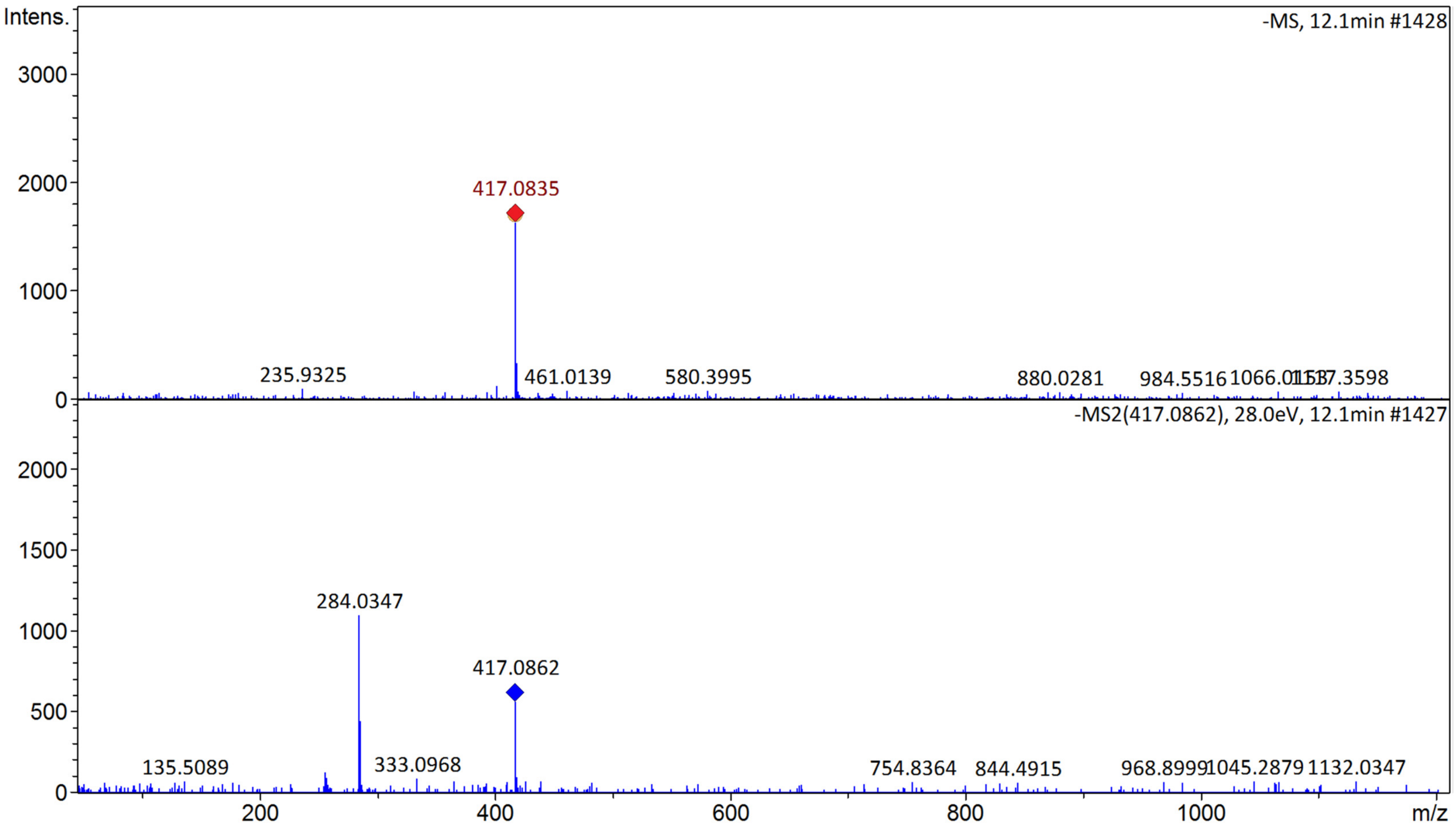

Figure S17
Peak at RT 20.6 min, negative ion ESI-MS spectra of *m/z* 285.0391 (kaempferol, **8**).

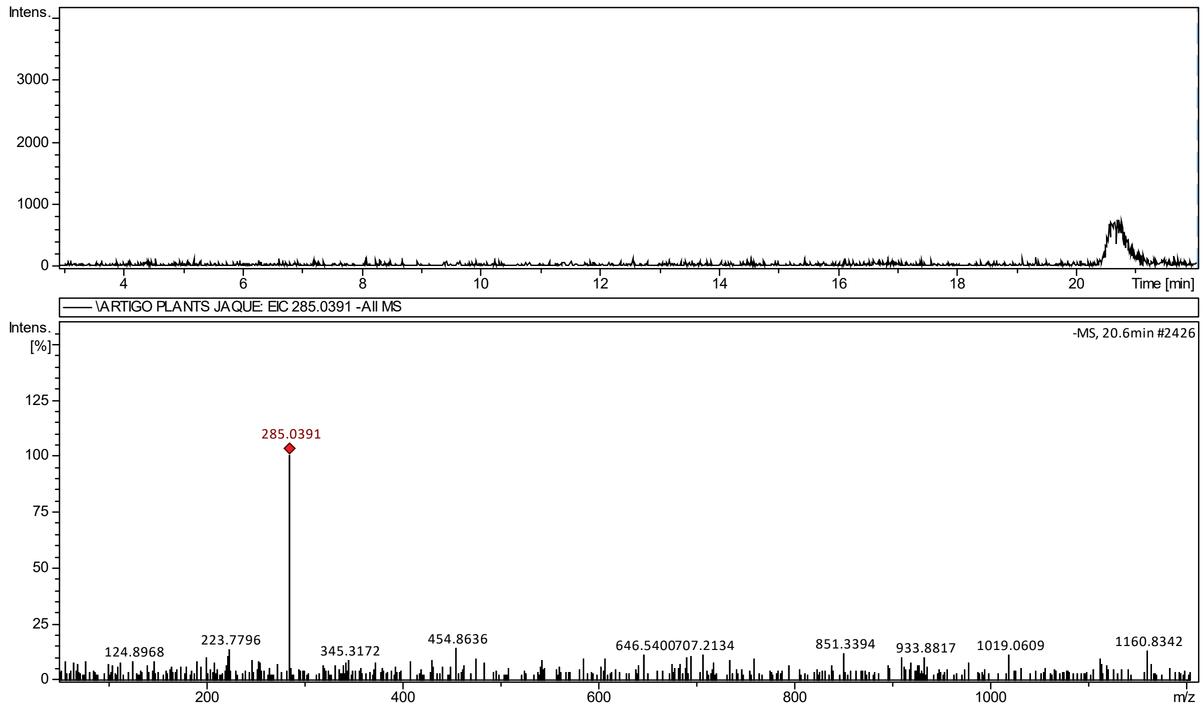

Supplement: Supplementary file 1 [file plants-14-00377-s001.zip › plants-3414092-supplementary.pdf]
